# Evaluation of Drug Trials in High-, Middle-, and Low-Income Countries and Local Commercial Availability of Newly Approved Drugs

**DOI:** 10.1001/jamanetworkopen.2021.7075

**Published:** 2021-05-05

**Authors:** Jennifer E. Miller, Michelle M. Mello, Joshua D. Wallach, Emily M. Gudbranson, Blake Bohlig, Joseph S. Ross, Cary P. Gross, Peter B. Bach

**Affiliations:** 1 Department of General Internal Medicine, Yale School of Medicine, New Haven, Connecticut; 2 Yale Program for Biomedical Ethics and Bioethics International, New Haven, Connecticut; 3 Stanford Law School, Freeman Spogli Institute for International Studies, Stanford University, Stanford, California; 4 Department of Health Research and Health Policy, Department of Medicine, Stanford University School of Medicine, Stanford, California; 5 Department of Environmental Health Sciences, Yale School of Public Health, New Haven, Connecticut; 6 Brigham and Women’s Hospital, Boston, Massachusetts; 7 Memorial Sloan Kettering Cancer Center, New York, New York

## Abstract

**Importance:**

Clinical research supporting US Food and Drug Administration (FDA) drug approvals is largely conducted outside the US.

**Objective:**

To characterize where drugs were tested for FDA approval and to determine how commonly and quickly these drugs received marketing approval in the countries where they were tested, both overall and by country income level and geographical region.

**Design, Setting, and Participants:**

This cross-sectional analysis of trials supporting FDA approval of novel drugs in 2012 and 2014, sponsored by large drug companies, did not involve human participants. The settings were the countries hosting trials supporting US drug approval. Data sources included Drugs@FDA, ClinicalTrials.gov, PubMed, Google Scholar, EMBASE, and drug regulatory agency websites. Data analysis was completed March through September 2020.

**Main Outcomes and Measures:**

The primary outcomes were the proportion of drugs approved for marketing in the countries where they were tested for FDA approval within 1, 2, 3, 4, and 5 years of FDA approval and the proportion of countries contributing participants to trials supporting FDA approvals receiving market access to the drugs they helped test within 1, 2, 3, 4, and 5 years of FDA approval.

**Results:**

In 2012 and 2014, the FDA approved 34 novel drugs sponsored by large companies, on the basis of a total of 898 trials, 563 of which had location information available. Each drug was tested in a median (interquartile range [IQR]) of 25 (18-37) unique countries, including a median (IQR) of 20 (13-25) high-income countries, 6 (4-11) upper-middle-income countries, and 1 (0-2) low-middle-income country. One drug was approved for marketing in all testing countries within 1 year of FDA approval and 15% (5 of 34 drugs) were approved in all testing countries within 5 years of FDA approval. Of the 70 countries contributing research participants for FDA drug approvals, 7% (5 countries) received market access to drugs they helped test within 1 year of FDA approval and 31% (22 countries) did so within 5 years. Access within 1 year occurred in 13% (5 of 39) of high-income countries, 0 of 22 upper-middle-income countries (0%), and 0 of 9 lower-middle-income countries (0%), whereas at 5 years access rates were 46% (18 of 39 countries), 9% (2 of 22 countries), and 22% (2 of 9 countries), respectively. Approvals were faster in high-income countries (median [IQR], 8 [0-11] months) than in upper-middle-income countries (median [IQR], 11 [5-29] months) or lower-middle-income countries (median [IQR], 17 [11-27] months) after FDA approval. Access was lowest in African countries.

**Conclusions and Relevance:**

These findings suggest that substantial gaps exist between where FDA-approved drugs are tested and where they ultimately become available to patients, raising concerns about the equitable distribution of research benefits at the population level.

## Key Points

**Question:**

How commonly are drugs commercially available in the countries where they were tested?

**Findings:**

This cross-sectional study found that 5 years after their approval in the US, 15% of novel drugs (5 of 34 drugs) were approved in all countries where they were tested; among 70 countries contributing research participants, 7% (5 countries) received market access to the drugs they helped test within 1 year of US approval and 31% (22 countries) did so within 5 years. Approvals were faster in high-income countries, and access was lowest in African countries.

**Meaning:**

These findings suggest that substantial gaps exist between where drugs are tested and where they become available to patients, raising concerns about the equitable distribution of research benefits.

## Teaser

This cross-sectional study examines where drugs were tested for US Food and Drug Administration approval and determines how commonly and quickly these drugs received marketing approval in the countries where they were tested, both overall and by country income level and geographical region.

## Introduction

In drug pricing policy discussions, an often heard refrain is that US taxpayers subsidize a substantial portion of drug research, particularly basic research,^1,2^ which benefits patients around the world,^3,4^ yet US drug prices are much higher than those in other countries.^5^ Not often mentioned is that clinical research supporting US medicine and vaccine approvals has been globalized and is largely conducted in other countries, increasingly lower-income countries.^6,7^ For heart failure trials, for example, participant enrollment from North America decreased from 43% in 2001 to 2004 to 27% in 2013 to 2016.^8^ The participation of patients in research conducted across international settings allows US patients to benefit from new medications.

This arrangement could, in theory, maximize benefits for all: the US contributes capital that other countries lack, while those countries contribute the human volunteers and workforce necessary to complete clinical trials expeditiously, and all enjoy the fruits of the research. Yet, for this to be true, the other countries would need to have access to the investigational products ultimately approved for marketing. Notably, the fact that a new drug receives approval from the US Food and Drug Administration (FDA) does not ensure market access in other countries. Pharmaceutical companies must decide to submit additional marketing approval applications in other countries.

To our knowledge, the rate at which market access is actually obtained in countries hosting trials, as well as the timing of such marketing approvals, has not been studied. Therefore, we sought to answer 3 questions. First, where were novel drugs approved by the FDA in 2012 and 2014 tested for approval? Second, how commonly and how quickly were these drugs approved for marketing in the countries where they were tested? Third, were there differences in how long it took for any marketing approvals to occur by country income level or geographical regions where testing occurred? Although marketing access does not guarantee that a patient can afford a medicine or vaccine or that there is a reasonably supply of a medical product, it is a critical precondition for access. Analyzing 563 trials for which location data were available, we explored the hypotheses that (1) many countries contributing research participants for FDA drug approvals would not have market access to those drug even 5 years after FDA approval, and (2) any approvals would be more common and faster in high-income than lower-income countries.

## Methods

This cross-sectional study did not undergo institutional review board review and informed consent was not needed or sought because it was not human subjects research, in accordance with 45 CFR §46. The Strengthening the Reporting of Observational Studies in Epidemiology (STROBE) reporting guideline for cross-sectional studies was used to ensure accurate reporting.

### Data Sources and Collection Methods

Using previously published methods and previously collected data,^9,10,11^ we identified all novel drugs sponsored by large companies approved by the FDA in 2012 or 2014 and all clinical trials supporting their FDA approval (ie, all clinical trials in the approved New Drug Application), along with trial characteristics, from Drugs@FDA (a publicly accessible database available through the FDA’s website containing records of FDA drug regulatory decisions). In keeping with previous methods, novel drugs were defined as new molecular entities and combination drugs with at least 1 new molecular entity. We selected approval years before 2015 to allow for 5-year follow-up of international drug approvals for our sample. In particular, we selected approval years 2012 and 2014 and drugs sponsored by large companies for convenience because we had already identified trials conducted for FDA approval of these drugs sponsored by large companies for other studies. Additionally, large companies are likely better able than smaller companies to bring products to market in multiple countries, because they have more resources. In accordance with our previously published methods,^9,10,11^ large companies were defined as the 20 largest companies measured by their market capitalization in the year of their product approval.

Using the same previously published methods,^9,10,11^ we matched these trials with trials registered in ClinicalTrials.gov, a clinical trial registry and database maintained by the National Library of Medicine, as well as corresponding publications in PubMed, Google Scholar, and EMBASE indexed journals. Using all sources, we recorded trial identification number, type (ie, interventional or observational), National Clinical Trial number, start date, primary completion date, number of enrolled participants, trial phase, sponsor, condition, approved indication, and trial country locations, among other characteristics. Discrepancies were reconciled through discussion and agreement.

We classified the drugs by orphan, accelerated approval, and FDA priority review status (ie, whether an FDA review was required to be completed within 6 instead of 10 months), using a previously described approach.^12,13^ We also categorized each drug’s initial indication using the World Health Organization’s Anatomical Therapeutic Classification system and then grouped these indications into 1 of 6 treatment areas: cancer; infectious disease; cardiovascular disease and diabetes; autoimmune, musculoskeletal, and dermatology; neurology; and psychiatry.^14^

After ascertaining the countries where each drug was tested to support FDA approval, 2 trained researchers (E.M.G. and B.B.) independently searched websites of the drug regulatory agencies for these countries (eg, European Medicines Agency [EMA], EudraPharm, and Health Canada) to determine whether each drug received marketing approval for any indication in each country where it was tested for FDA approval, resolving any discrepancies through discussion. If a drug was approved, one of these researchers (E.M.G.) went back to extract the approval date. Trials without location data were excluded from the sample (where this occurred, it was generally for phase 1 trials). Each country’s regulatory agency website contains a public searchable database of regulatory approval decisions. If regulatory agency websites were not in English, we used Google Translate to navigate and search the pages. Search terms used to locate and match a drug in such websites included drug name (both branded and generic names), trial and New Drug Application sponsor, FDA-approved indications, and other abstracted trial characteristics. An initial round of data abstraction to identify drug, trial characteristics, and regulatory approvals was conducted from October 2015 through April 2016, and a second search of regulatory agency websites was performed from February through March 2020 to identify regulatory approval dates and update data. This allowed an observation period of 5 years after FDA approval for each drug. If an approval was lacking a specific date, we counted it as approved within 5 years of FDA approval. Centralized marketing authorization processes were deemed to constitute marketing approval in all countries they covered; for example, an EMA marketing authorization allows the drug maker to market the drug in European Union Member States, Iceland, Liechtenstein, and Norway. We categorized a drug as approved in a country regardless of whether the company submitting the application was the same as the company that sponsored the trial (this distinction applied to only 1 drug).

We emailed each pharmaceutical company sponsoring a drug in our sample to ask whether they wanted to request any corrections in our data or tabulations and whether any drugs were submitted for marketing approval in any of our listed countries but not approved. Only 1 company provided approval information, which did not change our results.

### Outcome Measures

We determined the number (median and interquartile range [IQR]) of countries where each drug was listed as tested for FDA approval and report the median number of countries per drug overall, as well as by country national income group (high, upper-middle, lower-middle, and low income) using the World Bank historical classifications of the various economies in the year each tested drug was approved.^15^ We also determined whether each drug received marketing approval in each country where it was tested, other than the US, within 5 years after the FDA approval, and if so, on what date. We calculated the proportion and median number of drugs approved for marketing in each country, as well as in all countries and in each income group within 1, 2, 3, 4, and 5 years, respectively, of FDA approval. We excluded the US from these calculations because all drugs were tested and approved there.

We further determined the proportion of drugs approved for sale in tested countries at 1 and 5 years after FDA approval, by FDA priority review designation and by rare disease. FDA priority review designation was used as a proxy for a drug’s importance because a drug application with a priority review designation has been judged by the FDA to represent a substantial improvement in the safety or effectiveness of the treatment, diagnosis, or prevention of serious conditions, if approved.

Furthermore, we determined the proportion of countries contributing participants to trials supporting FDA approval of drugs that received market access to the drugs they helped test, within 1, 2, 3, 4, and 5 years of FDA approval. Market access rates are reported for the country cohort, across country income levels (high, upper-middle, and lower-middle income), and across 8 geographical regions (Africa, Western Europe, Eastern Europe, Asia, Middle East, Oceania, Latin America, and North America). We classified Russia as Eastern Europe, as opposed to Asia, because most of its population lives in Europe.

### Statistical Analysis

Results are presented by months and years after FDA approval. We conducted descriptive statistical analyses, including medians, IQRs, and proportions, using Excel spreadsheet software version 15.18 (Microsoft). Data analysis was completed March through September 2020.

## Results

### Characteristics of Novel Drugs, Sponsored by Large Companies

The FDA approved a total of 79 new novel drugs in 2012 and 2014 (48 in 2012 and 31 in 2014), of which 43% (34 drugs) were sponsored by large pharmaceutical companies (15 in 2012 and 19 in 2014). FDA applications for these drugs were based on a total of 898 trials (345 in 2012 and 553 in 2014). The 34 drugs were approved for a total of 28 unique indications. Neurology and psychiatry (10 of 28 drugs [29%]) and infectious disease (10 of 28 drugs [29%]) were the most common indications. One-half of the drugs (17 of 34 drugs [50%]) received priority review, and 12% (4 of 34 drugs) received accelerated approval. Approximately one-quarter (9 of 34 drugs [26%]) were designated as orphan products (Table).

**Table.** Characteristics of the Novel Drugs Sponsored by Large Companies and Approved by the US Food and Drug Administration in 2012 and 2014

Characteristic	Drugs, No. (%) (N = 34)
Approval year	
2012	15 (44)
2014	19 (56)
Treatment area	
Cancer and hematology	9 (26)
Infectious disease	10 (29)
Cardiovascular, diabetes, and hyperlipidemia	5 (15)
Autoimmune, musculoskeletal, and dermatology	3 (9)
Neurology and psychiatry	10 (29)
Other	6 (18)
Priority review, yes	17 (50)
Accelerated approval, yes	4 (12)
Orphan drug, yes	9 (26)
With public trial location data, yes	34 (100)
Trials with public location data (n = 898)	563 (63)

### Clinical Trial Locations

Trial location data were available for all 34 reviewed drugs but only 63% of trials (563 of 898 trials). Trials missing location data were generally unregistered, unpublished phase 1 trials. Our analyses are based on the 563 trials with location information.

**Figure 1.**
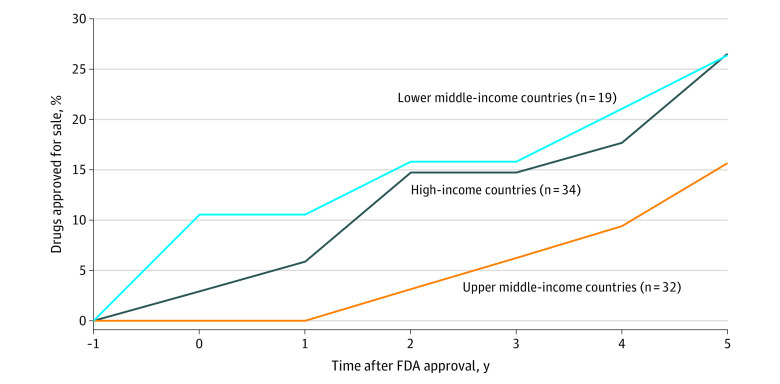
Percentage of Drugs Approved for Sale in All the Countries Where They Were Tested to Gain US Food and Drug Administration (FDA) Approval, by Income Level

Each drug was tested in a median (IQR) of 25 (18-37) unique countries in addition to the US, including a median (IQR) of 20 (13-25) high-income countries, 6 (4-11) upper-middle-income countries, and 1 (0-2) low-middle-income country. All 34 drugs in our sample (100%) were tested in at least 1 high-income country, 32 (94%) were tested in at least 1 upper-middle-income country, and 19 (56%) were tested in at least 1 lower-middle-income country. Only 1 drug, Sirturo (bedaquiline), a pulmonary multidrug-resistant tuberculosis treatment, was tested in a low-income country, Kenya (Figure 1 and eTable 1 and eTable 2 in the Supplement). Seventeen drugs were tested in South Africa.

**Figure 2.**
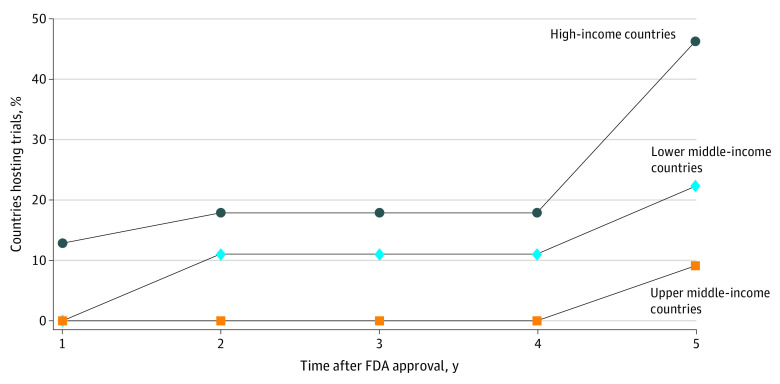
Percentage of Countries Hosting Trials for US Food and Drug Administration (FDA) Drug Approvals That Received Marketing Approval for Those Drugs Within 5 Years, by Income Level

Drugs were most commonly tested in Germany (91% [31 of 34 drugs]), followed by Austria (81% [30 of 34 drugs]), Spain and Canada (both 85% [29 of 34 drugs]), Poland (82% [28 of 34 drugs]), United Kingdom and Belgium (both 79% [27 of 34 drugs]), France (74% [25 of 34 drugs]), Italy (71% [24 of 34 drugs]), Sweden, Japan, and Brazil (all 3 at 68% [23 of 34 drugs]), and Russia, South Korea, and the Netherlands (all 3 at 62% [21 of 34 drugs]). See Figure 2 and eTable 3 in the Supplement for complete list of countries and the proportion of drugs for which testing took place within the country.

### Marketing Approval Rates and Timing at the Drug Level

Within 1 year of FDA approval, 3% of drugs (1 of 34 drugs) were approved for marketing in all countries where they were tested, and this proportion increased to 15% (5 of 34 drugs) when assessed within 5 years of FDA approval (eTable 1 in the Supplement). With regard to approval rates by country income levels, 6% of drugs (2 of 34 drugs) were approved in all the high-income countries where they were tested, 0% (0 of 32 drugs) were approved in upper-middle-income countries, and 11% (2 of 19 drugs) were approved in lower-middle-income countries within 1 year of FDA approval. These numbers increased to 26% (9 of 34 drugs), 16% (5 and 32 drugs), and 26% (5 of 19 drugs), respectively, when assessed at 5 years after FDA approval (Figure 1). Where drugs were approved, approvals occurring after FDA approval were faster in high-income countries (median [IQR], 9 [5-13] months) than in upper-middle-income countries (median [IQR], 14 [9-34] months) or lower-middle-income countries (median [IQR], 20 [13-26] months) (eTable 4, eTable 5, and eTable 6 in the Supplement).

No apparent differences were observed in market access rates for drugs with vs those without FDA priority review designations. Drugs for rare diseases were less likely to be approved than those for nonrare diseases across our all-country sample within 1 year (median [IQR], 52% [14%-71%] vs 66% [44%-75%] of drugs) and at 5 years (median [IQR], 76% [60%-87% vs 84% [68%-89%] of drugs) after FDA approval (eTable 7 in the Supplement).

### Marketing Approval Rates and Timing, on the Country Level

**Figure 3.**
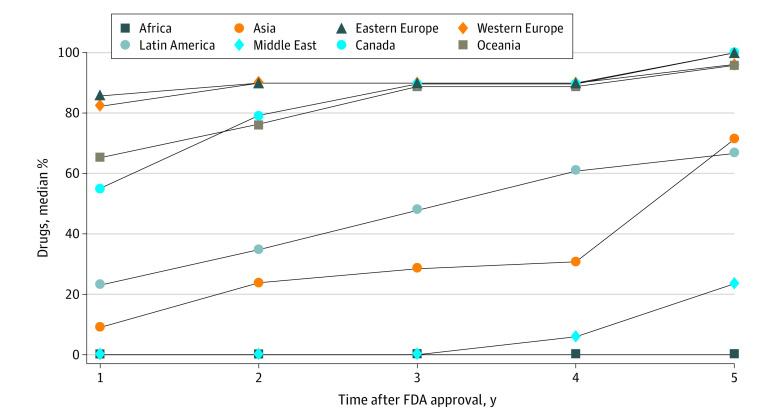
Percentage of Drugs Approved for Sale in the Countries Where They Were Tested for US Food and Drug Administration (FDA) Approval, by Geographical Regions

Of the 70 countries that contributed research participants for FDA drug approvals, 5 (7%) got market access to the drugs they helped test within 1 year after FDA approval and 22 (31%) did so at 5 years (eTable 4, eTable 5, and eTable 6 in the Supplement). Stratifying market access by country income level, we found that 13% of high-income countries (5 of 39 countries) got access within a year, compared with 0 upper-middle-income and lower-middle-income countries, to drugs they helped test. These numbers increased to 46% of high-income countries (18 of 39 countries), 9% of upper-middle-income countries (2 of 22 countries), and 22% of lower-middle-income countries (2 of 9 countries), when assessed at 5 years after FDA approval of drugs (Figure 3 and eFigure 1 in the Supplement).

By geographical regions, market access to medicines was lowest in African countries followed by Middle Eastern countries (Figure 3). Market access was highest in Canada and Eastern European countries within 5 years of FDA, followed by Western European countries and Oceania. Latin American and Asian countries fell toward the middle of the pack (Figure 3 and eFigure 2 in the Supplement).

## Discussion

A bedrock principle of research ethics is that the benefits and burdens of research should be shared equitably by the people affected by it.^16^A corollary is that to avoid exploitation, research should not ordinarily be conducted in a national population that does not stand to benefit from the knowledge to be gained or interventions to be developed.^17^ Despite the importance of this principle, little is known about the benefits provided to national populations participating in clinical research for FDA drug approvals.

To begin to fill this knowledge gap, we examined whether and when countries hosting trials supporting FDA approval of novel drugs get market access to such drugs and found that most drugs were generally not approved for marketing where they were tested. Only 1 drug was approved for marketing in all countries where it was tested within 1 year of FDA approval and 15% were approved within 5 years of FDA approval. Seven percent of countries hosting trials for FDA approvals had market access to the drugs they helped test within 1 year of FDA approval and 31% did so within 5 years. Marketing approvals were more likely in high-income than upper-middle-income countries. Where approved, drug approvals came more swiftly in high-income countries. Market access to medicines was lowest in Africa followed by the Middle East.

Studies focused on research equity—that is, achieving a fair distribution of research burdens and benefits—often center on the “don’t test, (but) do sell” problem^18^: drugs are often marketed to patient groups that were not well represented in the trials leading to their approval,^19,20^ including women,^21^ the elderly,^22,23,24,25,26^ and socioeconomically disadvantaged racial/ethnic groups.^27^ Our analysis focused on the mirror image of this problem, in which research sponsors include populations in research but do not secure marketing approval for them afterward. Whether the market access disparities found in this study are ethically problematic may be open to debate.

Research ethicists agree that populations from which research participants are drawn should stand to benefit from the research, but not on the type or quantity of benefit owed to these populations. In the bioethics literature, the fair benefits framework, for example, is generally understood as content neutral and as suggesting that research sponsors should engage each population in a transparent and collaborative process to define benefits. Under this procedural approach, provision of ancillary medical care or building a new local school could qualify as suitable research benefits.^28^ In contrast, proponents of the responsiveness principle often specify that communities should receive posttrial access to successful studied interventions, as stated in the Declaration of Helsinki. Embedded in this argument can be an expectation that trials are targeting the health needs and priorities of communities hosting trials.^29^ Critics of the fair benefits framework question, with good reason, how realistic it is to expect ad hoc local committees from developing countries to consistently negotiate fair benefits from large, multinational, for-profit companies sponsoring research.^30^

Some may argue that ensuring market access to all tested drugs approved in the US for countries supporting their testing is a misguided goal, in light of criticisms that the FDA approves costly drugs that do not represent a substantial therapeutic advancement over existing therapies. To address this issue, we note that our subanalysis of drugs with a priority review designation (ie, drugs judged by the FDA to represent a substantial improvement in the safety or effectiveness of the treatment, diagnosis, or prevention of serious conditions) found no apparent differences in market access rates from non–priority review drugs. Furthermore, we counter that whether a drug meets national standards for clinical use and reimbursement through national health programs is a decision for host countries to make, not companies developing drugs. That can occur only if drug makers file for local marketing approval. When companies do not seek marketing approval, they effectively supplant local decision-makers’ role; moreover, their decisions may be driven by market prospects, rather than a country’s best interest.

Others might wonder about the moral weight of national boundaries, asking whether it is not good enough for a drug tested in a lower-middle-income country to be commercially available in any other lower-middle-income country. We worry that such a standard of equitable benefit could exacerbate inequities, as more affluent patients within such countries have greater ability than poorer individuals to travel to other countries to access treatments.

Previous studies have evaluated factors associated with drug lags—that is, delays in drugs becoming available in markets. Although none of those studies focused on lags for countries hosting trials, their findings suggest that regulatory complexity and fragmentation (ie, the mosaic of regulations),^31,32^ slower regulatory review times,^33,34,35,36^ the presence of a drug manufacturer in a country,^37^ weak patent protections,^38^ and less profitable markets^39^ may be associated with drug lags and companies deprioritizing market access in countries hosting trials for FDA drug approvals. One study^30^ found lags of up to 4 to 7 years between the time medicines and vaccines were submitted for approval in a high-income country and market access was granted in the 20 sub-Saharan Africa countries with the lowest disease burden. Relatedly, our study found that drugs are generally not tested in sub-Saharan Africa (only Kenya and South Africa hosted trials for our drug sample, with 17 drugs tested in South Africa and 1 in Kenya). Studies have also found that orphan drugs approved in the US are less likely to be commercially available in other countries, such as China, because of high prices and varying reimbursement processes, supporting our own observations that rare disease drugs are less commercially available outside the US.^40,41^ This finding could raise questions about whether market access responsibilities are different for rare diseases, for example because patient populations are likely very small in each country.

Sponsors submitting drugs for FDA approval should, arguably, routinely secure product approvals in the countries were those drugs are tested. To encourage this practice, host-country governments could require that clinical trial agreements include a commitment to submit a marketing approval application in the country within a specified time after FDA or EMA approval. Such agreements should extend to business partners and successors of clinical trial sponsors, in case, for example, trial sponsors differ from registration sponsors. Companies should also consider adopting a policy that they will not test drugs on populations to whom they do not intend to sell the tested products. Groups of countries could also consider harmonizing regional drug marketing regulatory standards, requirements, and processes, as appropriate, to decrease fragmentation and complexities and to streamline access to products for their patient populations. Finally, regular and transparent tracking, auditing, and reporting on product registrations in countries hosting trials could better help advance access to medicines and vaccines globally.^42^ To aid in this process, it would be helpful to increase transparency around trial site locations.

### Limitations

This study has limitations. First, although we have trial location data for all drugs in our sample, location data were missing for approximately one-third of the trials that supported FDA approvals of our drug sample, generally the phase 1 trials. Second, our methods could not identify marketing applications that had been submitted but not yet approved. Furthermore, although our review process examined whether drugs were initially approved and subsequently withdrawn from the market, it is possible (although unlikely) that withdrawals may not have been listed on some countries’ regulatory agency websites. Third, although it would be valuable to know how quickly drug manufacturers submitted marketing applications in the countries where their trials were conducted, only information on the approval dates for those applications was available. Drug lags may be due to long approval times at regulatory agencies, delays in initiating applications, or both. Our analysis focuses on how much time elapses before consumers in the host countries gained access to the drug but cannot separate these 2 potential reasons for delayed access. We do not include secondary approvals or drugs that are not FDA approved but are approved in other jurisdictions. There is uncertainty about the representativeness of our results for products sponsored by smaller companies. Fourth, marketing approval does not guarantee that a patient can access a drug, for a range of reasons, including supply constraints, coverage decisions by health technology assessment organizations, and out-of-pocket cost and affordability issues. Nevertheless, registering a product for sale in a country is a critical precondition for patient access.

## Conclusions

In this cross-sectional study, we examined the clinical trials supporting novel drug approvals by the FDA in 2012 and 2014, sponsored by large companies, comparing in which countries the clinical trials took place and how long before, if ever, the products were marketed in those countries, by geographical regions and country income levels. Our study reveals substantial gaps between where drugs approved in the US are tested and where they ultimately become available to patients. To deliver on the promise of a fair bargain in drug testing between high- and low-income countries, it is essential that this gap be closed.

## Notes

Supplement. **eTable 1.** Proportion of Drugs Approved for Sale, Within 1 Year and 5 Years of FDA Approval, in Tested Countries, by Country Income Group **eTable 2. **List of Drugs and Their Pharmaceutical Company Sponsors Included in Our Study **eTable 3.** List of Countries Hosting Trials, Categorized by Region, Supporting Novel FDA Drug Approvals in 2012 and 2014, Sponsored by Large Companies **eTable 4.** Months After FDA Approval to Marketing Approval in High-Income Countries, by Drug **eTable 5.** Months After FDA Approval to Marketing Approval in Upper-Middle-Income Countries, by Drug **eTable 6.** Months After FDA Approval to Marketing Approval in Lower-Middle-Income Countries, by Drug **eTable 7.** Percentage of Drugs Approved for Sale at 1 and 5 Years After FDA Approval in Tested Countries, by FDA Review Status **eFigure 1.** Percentage of Countries Hosting Trials for FDA Drug Approvals That Received Marketing Approval for Those Drugs Within 7 Years, by Income Level **eFigure 2.** Percentage of Tested Drugs Approved for Sale in the Dominican Republic and Bahamas
