# Supplementary material for: Evaluation of Drug Trials in High-, Middle-, and Low-Income Countries and Local Commercial Availability of Newly Approved Drugs
Source: JAMA Netw Open. 2021 May 5;4(5):e217075. doi: 10.1001/jamanetworkopen.2021.7075 (PMC8100865; doi:10.1001/jamanetworkopen.2021.7075)
Supplement: Supplement. — eTable 1. Proportion of Drugs Approved for Sale, Within 1 Year and 5 Years of FDA Approval, in Tested Countries, by Country Income Group eTable 2. List of Drugs and Their Pharmaceutical Company Sponsors Included in Our Study eTable 3. List of Countries Hosting Trials, Categorized by Region, Supporting Novel FDA Drug Approvals in 2012 and 2014, Sponsored by Large Companies eTable 4. Months After FDA Approval to Marketing Approval in High-Income Countries, by Drug eTable 5. Months After FDA Approval to Marketing Approval in Upper-Middle-Income Countries, by Drug eTable 6. Months After FDA Approval to Marketing Approval in Lower-Middle-Income Countries, by Drug eTable 7. Percentage of Drugs Approved for Sale at 1 and 5 Years After FDA Approval in Tested Countries, by FDA Review Status eFigure 1. Percentage of Countries Hosting Trials for FDA Drug Approvals That Received Marketing Approval for Those Drugs Within 7 Years, by Income Level eFigure 2. Percentage of Tested Drugs Approved for Sale in the Dominican Republic and Bahamas [file jamanetwopen-e217075-s001.pdf]

Supplemental Online Content

Miller JE, Mello MM, Wallach JD, et al. Evaluation of drug trials in high-, middle-, and low-income countries and local commercial availability of newly approved drugs. *JAMA Netw Open*. 2021;4(5):e217075. doi:10.1001/jamanetworkopen.2021.7075

eTable 1. Proportion of Drugs Approved for Sale, Within 1 Year and 5 Years of FDA Approval, in Tested Countries, by Country Income Group
eTable 2. List of Drugs and Their Pharmaceutical Company Sponsors Included in Our Study
eTable 3. List of Countries Hosting Trials, Categorized by Region, Supporting Novel FDA Drug Approvals in 2012 and 2014, Sponsored by Large Companies
eTable 4. Months After FDA Approval to Marketing Approval in High-Income Countries, by Drug
eTable 5. Months After FDA Approval to Marketing Approval in Upper-Middle-Income Countries, by Drug
eTable 6. Months After FDA Approval to Marketing Approval in Lower-Middle-Income Countries, by Drug
eTable 7. Percentage of Drugs Approved for Sale at 1 and 5 Years After FDA Approval in Tested Countries, by FDA Review Status
eFigure 1. Percentage of Countries Hosting Trials for FDA Drug Approvals That Received Marketing Approval for Those Drugs Within 7 Years, by Income Level
eFigure 2. Percentage of Tested Drugs Approved for Sale in the Dominican Republic and Bahamas

This supplemental material has been provided by the authors to give readers additional information about their work.

© 2021 Miller JE et al. *JAMA Network Open.*

eTable 1: Proportion of Drugs Approved for Sale, Within 1 Year and 5 Years of FDA Approval, in Tested Countries, by Country Income Group[a].												
	All countries where drugs were:			High-income countries where drugs were:			Upper-middle-income countries where drugs were:			Low-middle-income countries where drugs were:		
Drug indication	Tested (# countries)	Approved within 1 year	Approved within 5 years	Tested (# countries)	Approved within 1 year	Approved within 5 years	Tested (# countries)	Approved within 1 year	Approved within 5 years	Tested (# countries)	Approved within 1 year	Approved within 5 years
Meningitis Vaccine, pediatrics	4	0 (0%)	0 (0%)	3	0%	0%	1	0%	0%	0	-	-
Basal cell carcinoma	12	0 (0%)	11 (92%)	10	0%	90%	2	0%	100%	0	-	-
Insomnia	24	1 (4%)	5 (21%)	17	6%	24%	6	0%	17%	1	0%	0%
TB	15	1 (7%)	8 (53%)	5	0%	60%	6	17%	67%	3	0%	100%
Gaucher Disease	9	1 (11%)	3 (33%)	7	14%	43%	1	0%	0%	1	0%	0%
Rheumatoid arthritis	45	5 (11%)	34 (76%)	26	8%	96%	16	19%	75%	3	0%	0%
Lung cancer	37	5 (14%)	28 (76%)	25	16%	100%	10	10%	30%	2	0%	0%
Type 2 Diabetes	43	31 (42%)	36 (84%)	27	85%	93%	12	67%	92%	4	0%	25%
Leukemia	35	15 (43%)	21 (60%)	23	61%	78%	10	10%	40%	2	0%	0%
Skin infection	18	8 (44%)	12 (67%)	11	55%	73%	6	17%	50%	1	100%	-
Thrombotic cardiovascular events	36	16 (44%)	20 (56%)	27	56%	67%	9	11%	22%	0	-	0%
Urinary and abdominal infections	31	15 (48%)	21 (68%)	16	75%	94%	11	27%	55%	4	0%	0%
Otitis Externa	2	1 (50%)	2 (100%)	2	50%	100%	0	-	-	0	-	-
Type 2 Diabetes	41	21 (51%)	31 (76%)	25	68%	92%	12	33%	58%	4	0%	0%
Gaucher Disease	29	15 (52%)	20 (69%)	16	81%	94%	11	18%	55%	2	0%	25%
Skin infection	21	12 (57%)	13 (62%)	15	60%	67%	4	75%	100%	2	0%	0%
Colorectal cancer	46	29 (63%)	38 (83%)	33	76%	97%	11	36%	64%	2	50%	100%
Multiple Sclerosis	39	25 (64%)	32 (82%)	25	80%	96%	12	33%	83%	2	100%	100%
Ovarian Cancer	29	19 (66%)	26 (90%)	20	70%	95%	8	50%	75%	1	0%	0%
Onychomycosis	3	2 (67%)	2 (67%)	2	100%	100%	1	0%	0%	0	-	-
Type 2 Diabetes	51	35 (69%)	42 (82%)	32	81%	97%	14	50%	71%	5	-	-
Renal cell carcinoma	23	16 (70%)	21 (91%)	20	80%	100%	2	0%	50%	1	-	-
Colon/rectal cancer	24	17 (71%)	21 (88%)	19	79%	95%	5	40%	80%	0	-	-
Leukemia	28	20 (71%)	23 (82%)	24	71%	83%	4	75%	75%	0	-	-
Breast cancer	43	31 (72%)	38 (88%)	26	88%	96%	15	47%	87%	2	50%	100%
Pulmonary Fibrosis	19	14 (74%)	19 (100%)	15	80%	100%	4	50%	100%	0	-	-
Cushing's Disease	23	17 (74%)	20 (87%)	17	94%	100%	6	17%	50%	0	-	-
Anti-coagulant	46	34 (74%)	39 (85%)	30	93%	96%	12	50%	75%	4	0%	25%
Arthritis; psoriasis	27	21 (78%)	24 (89%)	22	82%	95%	5	60%	60%	0	-	-
Constipation	17	14 (82%)	15 (88%)	13	85%	92%	4	75%	75%	0	-	-
HIV	19	16 (84%)	16 (84%)	15	93%	93%	4	50%	50%	0	-	-
Hepatitis C	21	18 (86%)	21 (100%)	20	90%	100%	1	0%	100%	0	-	-
Hepatitis C	26	24 (92%)	26 (100%)	22	95%	100%	4	75%	100%	0	-	-
Glaucoma	10	10 (100%)	10 (100%)	10	100%	100%	0	-	-	0	-	-
Proportion of drugs sold in 100% of countries where tested		1/34 (3%)	5/34 (15%)		2/34 (6%)	9/34 (26%)		0/32 (0%)	5/32 (16%)		2/19 (11%)	4/19 (26%)
Median (IQR)	25 (18-37)	64% (43-74)	83% (67-89)	20 (13-25)	78% (55-85)	95% (85-99)	6 (4-11)	30% (10-50)	66% (50-81)	1 (0-2)	0% (0-0)	0 (0-25)

[a] Sirturo was the only drug tested in a low-income country (Kenya). It was approved for sale there.

Sold in 100% of countries
Sold in 75-99% of countries
Sold in 50-74% of countries
Sold in 25-49% of countries
Sold in <25% of countries

© 2021 Miller JE et al. *JAMA Network Open.*

eTable 2. List of Drugs and Their Pharmaceutical Company Sponsors Included in Our Study							
**Drug *(Generic name)***	**Indication**	**Classification**	**Company**	**Company headquarter**	**Accelerated approval?**	**Orphan drug**	**Priority review**
Aubagio (*Teriflunomide)*	Multiple Sclerosis	Autoimmune, musculoskeletal, and dermatology	Sanofi	France	No	No	No
Belsomra (*Suvorexant)*	Insomnia	Neurology and psychiatry	Merck, Sharp Dohme	USA	No	No	No
Bosulif (*Bosutinib)*	Leukemia	Cancer and hematology	Pfizer	USA	No	Yes	No
Cerdelga (*Eliglustat)*	Gaucher Disease	GI/Met	Genzyme/ Sanofi	USA	No	Yes	Yes
Dalvance *(Dalbavancin)*	Skin infection	Infectious Diseases	Durata/ Allergan[1]	Ireland	No	No	Yes
Elelyso (*Taliglucerase Alfa)*	Gaucher Disease	GI/Met	Pfizer	USA	No	Yes	No
Eliquis (*Apixaban)*	Anti-coagulant	Cardiovascular, diabetes, and hyperlipidemia	BMS	USA	No	No	Yes
Erivedge (*Vismodegib)*	Basal cell carcinoma	Cancer and hematology	Genentech/ Roche[2]	USA	No	No	Yes
Esbriet (*Pirfenidone)*	Pulmonary Fibrosis	Other	Genentech/ Roche[2]	USA/ Switzerland	No	Yes	Yes
Farxiga (*Dapagliflozin)*	Type 2 Diabetes	Cardiovascular, diabetes, and hyperlipidemia	Astrazeneca	United Kingdom	No	No	No
Harvoni *Ledipasvir/sofosbuvir)*	Hepatitis C	Infectious Diseases	Gilead	USA	No	No	Yes
Inlyta (*Axitinib)*	Renal cell carcinoma	Cancer and hematology	Pfizer	USA	No	No	No
Invokana (*Canagliflozin)*	Type 2 Diabetes	Cardiovascular, diabetes, and hyperlipidemia	Janssen/ J&J	USA	No	No	No
Jublia (*Efinaconazole)*	Onychomycosis	Infectious Diseases	Dow /Valeant	USA	No	No	No
Lynparza (*Olaparib)*	Ovarian cancer	Cancer and hematology	Astrazeneca	United Kingdom	Yes	Yes	Yes
MenHibrix (*Haemophilus b and Meningococcal Vaccine)*	Meningitis vaccine, pediatrics	Infectious Diseases	GSK	United Kingdom	No	No	No
Movantik (*Naloxegol)*	Constipation	GI/Met	Astrazeneca	United Kingdom	No	No	No
Otezla (*Apremilast)*	Arthritis; psoriasis	Autoimmune, musculoskeletal, and dermatology	Celgene	USA	No	No	No
Perjeta (*Pertuzumab)*	Breast cancer	Cancer and hematology	Genentech/ Roche[2]	USA/ Switzerland	No	No	Yes
Signifor (*Pasireotide)*	Cushing's Disease	GI/Met	Novartis	Switzerland	No	Yes	No
Sirturo (*Bedaquiline)*	TB	Infectious Diseases	J&J	USA	Yes	Yes	Yes
Sivextro (*Tedizolid)*	Skin infection	Infectious Diseases	Cubist/ Merck	USA	No	No	Yes
Stivarga (*Regorafenib)*	Colon/rectal cancer	Cancer and hematology	Bayer	Germany	No	No	Yes
Stribild (*Cobicistat/ Elvitegravir/ Emtricitabine/ Tenofovir)*	HIV	Infectious Diseases	Gilead	USA	No	No	No
Viekira Pak (*Dasabuvir, Ombitasvir, Paritaprevir, Ritonavir)*	Hepatitis C	Infectious Diseases	Abbvie	USA	No	No	Yes
Xeljanz (*Tofacitinib)*	Rheumatoid arthritis	Autoimmune, musculoskeletal, and dermatology	Pfizer	USA	No	No	No
Xigduo (*Dapagliflozin-Metformin)*	Type 2 Diabetes	Cardiovascular, diabetes, and hyperlipidemia	Astrazeneca AB	Sweden	no	no	no
Xtoro (*Finafloxacin Otic Suspension)*	Otitis Externa	Infectious Diseases	Alcon/ Novartis	Switzerland	No	No	Yes
Zaltrap (*Ziv-aflibercept)*	Colorectal cancer	Cancer and hematology	Sanofi	France	No	No	Yes
Zerbaxa (*Ceftolozane-Tazobactam)*	Urinary and abdominal infections	Infectious Diseases	Cubist/ Merck	USA	No	No	Yes
Zioptan (*Tafluprost Ophthalmic)*	Glaucoma	Other	Merck	USA	No	No	No
Zontivity (*Vorapaxar)*	Thrombotic cardiovascular events	Cardiovascular, diabetes, and hyperlipidemia	Merck Sharp Dohme	USA	No	No	No
Zydelig (*Idelalisib)*	Leukemia	Cancer and hematology	Gilead	USA	Yes	Yes	Yes
Zykadia(*Ceritinib)*	Lung cancer	Cancer and hematology	Novartis	Switzerland	Yes	Yes	Yes

[1]Allergen acquired Durata. Allergan's global headquarter is listed.
[2] Genentech/Roche: Genentech is a member of the Roche group. Genentech's global headquarter (GHQ) is in the US. Roche's GHQ is in Switzerland.
[3] Xigduo was acquired from Bristol-Myers Squibb on 01-Feb-2014

© 2021 Miller JE et al. *JAMA Network Open.*

eTable 3. List of Countries Hosting Trials, Categorized by Region, Supporting Novel FDA Drug Approvals in 2012 and 2014, Sponsored by Large Companies (Number of Drugs = 34)

Drug	Region	# of drugs tested
Germany	Western Europe	31
Austria	Western Europe	30
Spain	Western Europe	29
Belgium	Western Europe	27
UK	Western Europe	27
France	Western Europe	25
Italy	Western Europe	24
Sweden	Western Europe	23
Netherlands	Western Europe	21
Switzerland	Western Europe	17
Denmark	Western Europe	16
Finland	Western Europe	15
Portugal	Western Europe	14
Greece	Western Europe	11
Norway	Western Europe	9
Ireland	Western Europe	8
Iceland	Western Europe	1
Luxemburg	Western Europe	1
Slovenia	Western Europe	1
Australia	Oceania	18
New Zealand	Oceania	12
Canada	North America	29
Puerto Rico	North America	12
Dominican Republic	North America	2
Bahamas	North America	1
Israel	Middle East	20
Turkey	Middle East	17
Jordan	Middle East	2
Lebanon	Middle East	2
Brazil	Latin America	23
Mexico	Latin America	20
Argentina	Latin America	17
Peru	Latin America	15
Colombia	Latin America	13
Chile	Latin America	10
Costa Rica	Latin America	3
Guatemala	Latin America	2
Panama	Latin America	1
Venezuela	Latin America	1
Paraguay	Latin America	1
Poland	Eastern Europe	28
Russia	Eastern Europe	21
Hungary	Eastern Europe	20
Czech Republic	Eastern Europe	16
Romania	Eastern Europe	15
Slovakia	Eastern Europe	14
Bulgaria	Eastern Europe	13
Ukraine	Eastern Europe	13
Croatia	Eastern Europe	9
Estonia	Eastern Europe	9
Latvia	Eastern Europe	7
Lithuania	Eastern Europe	7
Serbia	Eastern Europe	3
Bosnia	Eastern Europe	2
Georgia	Eastern Europe	1
Moldova	Eastern Europe	1
Japan	Asia	23
Korea	Asia	21
China	Asia	18
Taiwan	Asia	15

© 2021 Miller JE et al. *JAMA Network Open.*

Singapore	Asia	13
India	Asia	13
Thailand	Asia	11
Hong Kong	Asia	8
Philippines	Asia	7
Malaysia	Asia	5
Vietnam	Asia	3
South Africa	Africa	17
Tunisia	Africa	2
Egypt	Africa	1

© 2021 Miller JE et al. *JAMA Network Open.*

eTable 4. Months After FDA Approval to Marketing Approval in High-Income Countries, by Drug (Total Number of Countries Is 39)

	High-income country																		
**Drug**	**At**	**Au**	**Bs**	**Be**	**Ca**	**Cl**	**Hr**	**Cz**	**Dk**	**Ee**	**Fi**	**Fr**	**De**	**Gr**	**Hk**	**Is**	**Ie**	**Il**	**It**
Aubagio	11	2		11	14	11		11	11	11	11	11	11	11					11
Belsomra	27				56														3
Bosulif	19	6		6	19	51					6	6	6						6
Cerdelga	6	5			34		5					5	5	5				16	5
Dalvance					51		9			9			9						
Elelyso	24				28													8	
Eliquis	0*	0*		0*	0*	7		0*	0*		0*	0*	0*	0*	0*			1	0*
Erivedge	16			18								18	18					12	
Esbriet	16			0*	0*		0*					0*	0*				0*	15	0*
Farxiga	0*	0*		0*	12	0*			0*		0*	0*	0*		10		0*	36	0*
Harvoni	7	1		1	0			1		1		1	1						1
Inlyta	6	8		8	7			8				8	8	8			8		8
Invokana	4	0		0	7			0	0	0	0	0	0	0	16	0		14	
Jublia					1														
Lynparza	13			0	17			0	0	0		0	0					37	0
MenHibrix																			
Movantik	16			0*	11		0*	0*					0*						
Otezla	12	10		10	8			10	10	10	10	10	10						10
Perjeta	11	9		9	11		9				9	9	9	9				8	9
Signifor	11			0*	11				0*		0*	0*	0*	0*				13	0*
Sirturo										15			15						
Sivextro					16			9					9						
Stivarga	14			11	7			11			11	11	11		26			28	11
Stribild	6	9		9	4				9			9	9						9
Viekira Pak	7	1		1	0*			1	1			1	1				1	0*	1
Xeljanz	27	50		50	19	14	50	50	50		50	50	50	50			50		50
Xigduo	0*	0*		0*	16	16		0*	0*		0*	0*	0*		35		9	51	0*
Xtoro					51														
Zaltrap	8	6		6	21	16	6	6	6	6	6	6	6	6				65	6
Zerbaxa	11			9		25	9			9			9						
Zioptan											0*	0*	0*					0*	0*
Zontivity		8		8	30			8	8		8	8	8						8
Zydelig	7	2		2	9		2	2	2			2	2	2			2		2
Zykadia	23	13		13	12				13			13	13	13	20		13	9	13

	Country																			
**Drug**	**Jp**	**Kr**	**Lv**	**Lt**	**Lu**	**Nl**	**Nz**	**No**	**Po**	**Pt**	**Pr**	**Ru**	**Sg**	**Sk**	**Sl**	**Sp**	**Se**	**Ch**	**Tw**	**UK**
Aubagio				11		11		14	11	11		75		11		11	11			11
Belsomra	3											28								
Bosulif	24		6	6		6		9	6							6	6			6
Cerdelga	7					5				5						5	5			5
Dalvance			9	9					9					9			9			9
Elelyso																				
Eliquis	0					0*	6	0*	0*	0*	0	0*	0	0*		0*	0*		8	0*
Erivedge						18			18							18				18
Esbriet	0*						20		0*				42			0*		11		0*
Farxiga	2					0*			0*		0		34	0*		0*	0*	7	12	0*

© 2021 Miller JE et al. *JAMA Network Open.*

Harvoni	9	19				1	1		1		0					1	1	2	14	1
Inlyta									8				14	8		8	8		10	8
Invokana	8		0	0	0	0	12	63	0	0	0	17	71	0		0	0	2		0
Jublia	1																			
Lynparza	37	8				0			0					0		0	0	13		0
MenHibrix																				
Movantik									0*		0			0*		0*	0*			0*
Otezla				10		10	32		10			30		10		10		10	42	10
Perjeta	12	11	9	9		9	12		9	9		38	20			9	9	2	12	9
Signifor						0*			0*	0*						0*	0*	0*		0*
Sirturo			15																	
Sivextro			9						9			23		9		9				
Stivarga	6					11			11	11			9			11		5		11
Stribild						9				9	0					9	9			9
Viekira Pak	9					1	8		1	1	0	0*				1	1	16		1
Xeljanz	4	73							50		0		24	50		50	50		13	50
Xigduo						0*			0*		0	48		0*		0*	0*		32	0*
Xtoro											0									
Zaltrap	29	6				6	28	9	6	6	0	23	24	6		6	6	2	11	6
Zerbaxa		28	9	9		10		9				45		9	9	9				
Zioptan								0*	0*							0*	0*	0*		
Zontivity						8	34		8	8	0					8	8	28		8
Zydelig							19		2	2			19			2	2		25	2
Zykadia	23	9				13		15	13	13		47	12			13	13	16	20	13

Approved for marketing before FDA approval (0*)
Approved for marketing 0-1 years after FDA approval
Approved for marketing 1-2 years after FDA approval
Approved for marketing >2 years after FDA approval
Approved for marketing, but approval date unknown
Tested but not approved for marketing in country
White = not tested in that country
Median time to approval, excluding countries with unknown approval dates = 8 months (IQR 0-11)
At = Austria; Au = Australia; Be = Belgium; Bs = Bahamas; Ca = Canada; Cl = Chile, Hr = Croatia; Cz = Czech Republic; Dk = Denmark; Ee Estonia; Fi = Finland; Fr = France; De = Germany; Gr = Greece; Hk = Hong Kong; Is= Iceland; Ie = Ireland; Il = Israel; It = Italy; Jp = Japan; Kr = Korea; Lt = Latvia; Lt = Lithuania; Lu = Luxemburg; Nl = Netherlands; Nz = New Zealand; No = Norway; Po = Poland; Pt = Portugal; Pr = Puerto Rico; Ru = Russia; Sg = Singapore; Sk = Slovakia; Sl = Slovenia; Sp = Spain; Se = Sweden; Ch = Switzerland; T = Taiwan; UK = United Kingdom

© 2021 Miller JE et al. *JAMA Network Open.*

eTable 5. Months After FDA Approval to Marketing Approval in Upper-Middle-Income Countries, by Drug (Total Number of Countries = 22)

	**Upper-middle income country**																					
**Drug**	**Ar**	**Bo**	**Br**	**Bu**	**Cn**	**Co**	**CR**	**DR**	**Hu**	**Jo**	**Lb**	**My**	**Mx**	**Pa**	**Pe**	**Ro**	**Srb**	**SA**	**Th**	**Tn**	**Tr**	**Vz**
Aubagio	6		72	11	70	16			11				15			11						
Belsomra													28									
Bosulif	13					65			6				31									
Cerdelga	34		52	5									14			5	61					
Dalvance				9					9							9						
Elelyso																						
Eliquis	6		55	0*		0*			0*			13			0*	0*						
Erivedge			14												24							
Esbriet			10										32		10							
Farxiga	0*		20	0*	38	0*			0*				9		10	0*		0*			71	
Harvoni					50																	
Inlyta			39																			
Invokana	18		10	0		0*	57					45			0*	0						
Jublia																						
Lynparza	20		9						0				10		22	0						
MenHibrix																						
Movantik				0*					0*							0*						
Otezla				10					10							10						
Perjeta	12	83	12				67		9				3		21	9	18		19		44	
Signifor	10		33										26									
Sirturo			79		48										17			0				
Sivextro			13						9				22									
Stivarga	10		46						11													
Stribild													8						9			
Viekira Pak									1				1			1					44	
Xeljanz	6	66	25	50	52	65	54		50				11		4	50						
Xigduo	22		27	0*					0*				2		29	0*						
Xtoro																						
Zaltrap	46		46	6					6				21									
Zerbaxa	27		19	9					9				17			9	43					
Zioptan																						
Zontivity	26								8													
Zydelig				2					2							2						
Zykadia	15								13				10									

Approved for marketing before FDA approval
Approved for marketing 0-1 years after FDA approval
Approved for marketing 1-2 years after FDA approval
Approved for marketing >2 years after FDA approval
Approved for marketing, but approval date unknown
Tested but not approved for marketing in country
White = not tested in that country
Median time to approval, excluding countries with unknown approval dates = 11 months (IQR 5-29)
Ar = Argentina; Bo = Bosnia; Br = Brazil; Bu = Bulgaria = Cn = China; Co = Columbia; CR = Costa Rica; DR = Dominican Republic; Hu = Hungary; Jo = Jordan; Lb = Lebanon; My = Malaysia; Mx = Mexico; Pa = Panama; Pe = Peru; Ro = Romania; Srb = Serbia; SA = South Africa; Th = Thailand; Tn = Tunisia; Tr = Turkey; Vz = Venezuela

© 2021 Miller JE et al. *JAMA Network Open.*

eTable 6. Months After FDA Approval to Marketing Approval in Lower-Middle-Income Countries, by Drug (Total Number of Countries = 9)

	Low-middle income country								
**Drug**	**Eg**	**Ge**	**Gt**	**In**	**Md**	**Py**	**Ph**	**Ua**	**Vn**
Aubagio								11	
Belsomra									
Bosulif									
Cerdelga									
Dalvance									
Elelyso									
Eliquis									
Erivedge									
Esbriet									
Farxiga				26					
Harvoni									
Inlyta									
Invokana			0*	17				27	
Jublia									
Lynparza								11	
MenHibrix									
Movantik									
Otezla									
Perjeta			13				16		
Signifor									
Sirturo				25			22	66	
Sivextro								9	
Stivarga									
Stribild									
Viekira Pak									
Xeljanz									
Xigduo				36					
Xtoro									
Zaltrap									
Zerbaxa									
Zioptan									
Zontivity									
Zydelig									
Zykadia									

Approved for marketing before FDA approval
Approved for marketing 0-1 years after FDA approval
Approved for marketing 1-2 years after FDA approval
Approved for marketing >2 years after FDA approval
Approved for marketing, but approval date unknown
Tested but not approved for marketing in country
White = not tested in that country
Median time to approval, excluding countries with unknown approval dates = 17 months (IQR 11-27)
Eg = Egypt; Ge = Georgia; Gt = Guatemala; In = India; Md = Moldova; Py = Paraguay; Ph = Philippines; Ua = Ukraine; Vn = Vietnam

© 2021 Miller JE et al. *JAMA Network Open.*

eTable 7. Percentage of Drugs Approved for Sale at 1 and 5 Years After FDA Approval in Tested Countries, by FDA Review Status

	No. drugs (%)	All countries where tested, Median (IQR)		High-income countries, Median (IQR)		Upper-middle-income countries, Median (IQR)		Low-middle-income countries, Median (IQR)	
		Approved within 1 year	Approved within 5 years	Approved within 1 year	Approved within 5 years	Approved within 1 year	Approved within 5 years	Approved within 1 year	Approved within 5 years
**ALL drugs**	34 (100)	64% (43-74)	83% (67-89)	78% (55-85)	95% (85-99)	30% (10-50)	66% (50-81)	0% (0-0)	0% (0-25)
**Drugs w priority review designation**	17 (50)	65% (47-73)	85% (69-92)	75% (55-81)	95% (90-100)	38% (17-50)	75% (62-100)	0% (0-38)	25% (0-100)
**Drugs w no priority review designation (standard review)**	17 (50)	64% (42-74)	82% (60-87)	80% (56-85)	93% (78-97)	18% (0-50)	50% (21-72)	0% (0-0)	0% (0-6)
**Rare disease drugs**	9 (26)	52% (14-71)	76% (60-87)	70% (16-80)	94% (78-100)	17% (10-50)	55% (40-75)	0% (0-0)	0% (0-19)
**Non rare diseases**	25 (74)	66% (44-75)	84% (68-89)	80% (56-88)	95% (92-97)	33% (6-50)	71% (50-85)	0% (0-50)	0% (0-63)

© 2021 Miller JE et al. *JAMA Network Open.*

eFigure 1. Percentage of Countries Hosting Trials for FDA Drug Approvals That Received Marketing Approval for Those Drugs Within 7 Years, by Income Level*

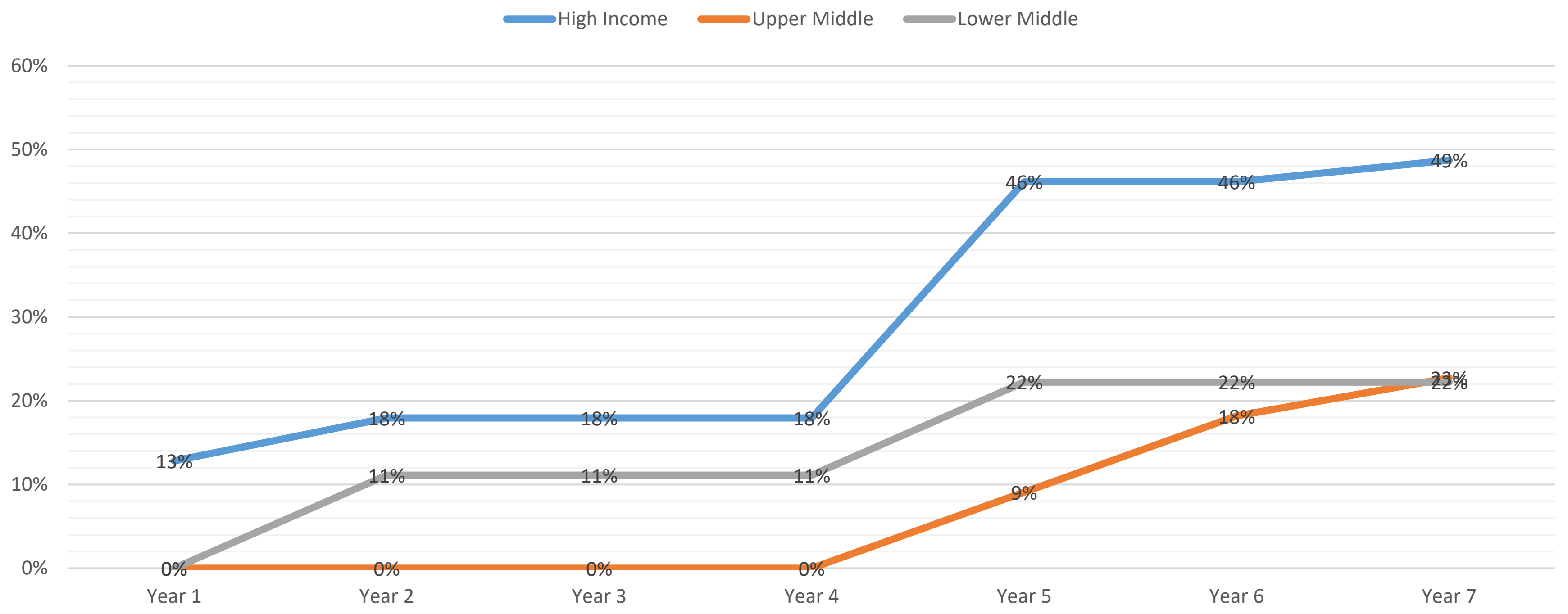

*Note: Data are incomplete for years 6 and 7 for drugs approved by the FDA in 2014, as not enough time has elapsed to capture all country approvals.

© 2021 Miller JE et al. *JAMA Network Open.*

eFigure 2. Percentage of Tested Drugs Approved for Sale in the Dominican Republic and Bahamas

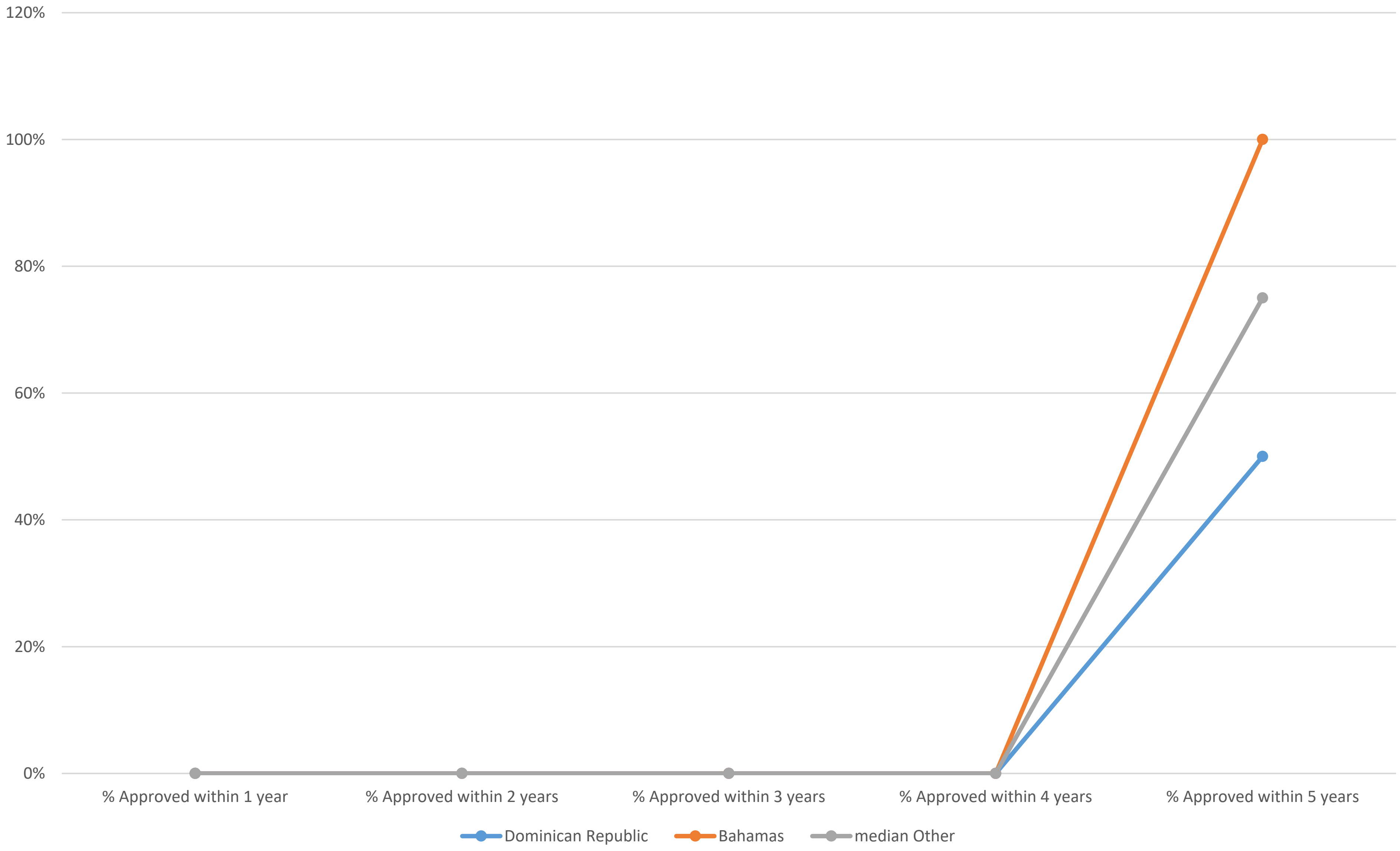

© 2021 Miller JE et al. *JAMA Network Open.*
